# Oral diuretic activity of hot water infusion of Sri Lankan black tea (**Camellia sinensis** L.) in rats

**DOI:** 10.4103/0973-1296.71788

**Published:** 2010

**Authors:** K. R. W. Abeywickrama, W. D. Ratnasooriya, A. M. T. Amarakoon

**Affiliations:** *Analytical Laboratory, Sri Lanka Tea Board, Colombo, Sri Lanka*; 1 *Department of Zoology, University of Colombo, Sri Lanka*; 2 *Biochemistry Division, Tea Research Institute, Talawakelle, Sri Lanka*

**Keywords:** Black tea, Camellia sinensis, diuretic activity, electrolytes, agroclimatic elevation, urine output

## Abstract

**Background:**

Black tea [**Camellia sinensis** (L.) O. Kuntze (family: Theaceae)] has been used by Sri Lankan traditional practitioners to promote diuresis. However, the type and grade of tea is not specified.

**Materials and Methods:**

This study investigates the diuretic activity of black tea infusion (BTI) in rats using Broken Orange Pekoe Fannings (BOPF) grade from major agroclimatic elevations: high-, mid-, and low-grown. Different concentrations of BTI, furosemide (positive control), and water (vehicle) were orally administered to starved (18 h) male rats (n = 9/group), then hydrated. Acute and chronic (28 days) diuretic activities were assessed by measuring cumulative urine output at hourly intervals for 6 h. Electrolyte levels (Na^+^, K^+^, Ca^2+^, H^+^, Cl^−^, HCO_3_^−^), pH, osmolarity of urine, and glomerular filtration rate (GFR) of treated rats were determined.

**Results:**

Administration of BTI induced a significant (*P* < 0.05) and dose-dependent diuretic activity, which varied with the tea produced in different agroclimatic elevations. Diuretic activity had a rapid onset (1^st^ h), peaked at 2^nd^ h and maintained up to 4^th^ h (except the low dose). Furthermore, there was a dose-dependent increase in micturition frequency, which peaked at 2^nd^ h. A close association between the caffeine content of tea and diuretic activity was evident. BTI-induced diuresis was accompanied with an increased urine Na^+^ level and GFR. The diuretic activity of BTI was mediated via multiple mechanisms: inhibition of both aldosterone secretion (with increased Na^+^/K^+^ ratio) and carbonic anhydrase [with decreased Cl^−^/(Na^+^ + K^+^) ratio] and via thiazide type of diuretic action (evaluated with increased Na^+^/Cl^−^ ratio).

**Conclusion:**

The Sri Lankan BOPF grade black tea possesses mild oral diuretic activity whose efficacy differs with the agroclimatic elevation of production. Furthermore, it supports the traditional claim that the black tea acts as a diuretic.

## INTRODUCTION

After water, brewed tea is the most consumed beverage in the world. Today, about 3–5 billion cups of tea is drunk daily by humans.[1] Tea is manufactured from the uppermost 2 leaves and the bud of **Camellia sinensis** (L.) O. Kuntze (Family: Theaceae) plant. There are 3 main types of made tea: green tea (produced by unfermented or unaerated process), oolong tea (produced by partially aerated process), and black tea (produced by fully aerated process).[1] The majority of global tea drinkers (about 78%) prefer to consume black tea.[2] Drinking of black tea is recommended in Sri Lankan traditional medicine to promote urinary flushing,[3] possibly due to its diuretic effect.[4] Induced diuresis is used clinically in medicine to reduce blood pressure and edema.[5] In black tea, diuresis is claimed to be mediated via caffeine, catechins, thearubigins and theaflavins.[1,4] Recently, Dust grade of high-grown Sri Lankan black tea was shown to posses mild diuretic activity.[4] It is well recognized that the phytochemical composition of black tea infusion (BTI) and its pharmaco-therapeutic properties vary with several factors, including agroclimatic elevation of production and size of the manufactured tea particles.[6,7] Thus, a possibility exists that diuretic potential of black tea could also vary depending on these factors.

Therefore, the aim of this study was to investigate the diuretic potential of BTI of Broken Orange Pekoe Fannings (BOPF) grade (larger particle size than Dust and very popular among tea bag consumers) orthodox black tea from major agroclimatic elevations in Sri Lanka: high-grown (above 1200 m, average mean sea level); mid-grown (between 600 and 1200 m, amsl); and low-grown (below 600 m, amsl) in rats.

## MATERIALS AND METHODS

### Black tea samples

Fresh BOPF grade black tea collected in May 2006 from randomly selected tea factories in major agroclimatic elevations in Sri Lanka: high-grown (St. Coombs Estate tea factory, Talawakelle), mid-grown (Dotaloya Estate, Kandy), and low-grown (Yatideriya Estate, Kegalle) were used. Drawn tea samples (1 kg of each) were packed in moisture-proof bags and stored at −20°C until use. The composition of true to size particles defined for the BOPF grade tea was determined using sieve shaker (Retsch AS 200, Retsch GmbH, Haan, Germany) and standard set of sieves.[8] Typical characters belonging to elevations were assessed organoleptically by professional tea tasters.[9]

### Experimental animals

Adult male Wistar rats weighing 200–225 g were used. They were maintained in the animal house at the Department of Zoology, University of Colombo, Sri Lanka, under standardized conditions (temperature at 28°C–31°C; average photo period of 12 h day light/dark cycle and relative humidity: 50%–55%), with food pellets (Ceylon Grain Elevators, Colombo, Sri Lanka) and water available *ad libitum*. All animal experiments were conducted in accordance with the internationally accepted laboratory animal use and care, and guidelines[10] and obtained clearance under the rules of the Department of Zoology, Faculty of Science, University of Colombo, Sri Lanka, for animal experimentations.

### Dosage of oral treatment of BTI

The BTI was freshly prepared, according to the international standards[9] [yield of tea solids (w/w) of BTI: 360, 430, and 440 g/kg for high-, mid-, and low-grown, respectively]. Three different doses of high-grown BTI: 300, 600, and 2400 mg/kg, body weight of rat (low, mid, and high doses, respectively) and single doses (2400 mg/kg) of mid-grown and low-grown BTI were made (equivalent to 1½, 3, and 12 cups, respectively: one cup is 170 ± 10 mL).

### Evaluation of acute diuretic activity

Diuretic activity was assessed as described previously.[4] Briefly, 63 rats were starved for 18 h and their urinary bladders were emptied by gentle compressions of the pelvic area and by pull of tails. Thereafter, 15 mL of isotonic saline (NaCl, 0.9% w/v) was orally administered by gastric intubation into these rats to inflict a uniform water load. Forty-five minutes later, these rats were randomly divided into 7 equal groups (n = 9/group) and orally administered with either water (vehicle: negative control) or different doses of BTI or furosemide (13 mg/kg body weight; standard diuretic drug: positive control) in the following manner: Group (1): water; Group (2), (3), and (4): low, mid, and high doses of high-grown BTI, respectively; Group (5): high dose of mid-grown BTI; Group (6): high dose of low-grown BTI; and Group (7): furosemide. Thereafter, each rat was placed individually in a metabolic cage and the cumulative urine output at hourly intervals was measured for 6 h. During this period, the frequency of urination was observed continuously. Furthermore, during this period no water and food was given to these animals. Concentrations of electrolytes in the urine: Na^+^ and K^+^ (Flame photometer: “Jencon PFP 7”; Jencons Scientific Limited, Bedfordshire, UK), Ca^2+^ (Atomic Absorption Spectrophotometer: “GBC 902”; GBC Scientific Inc, Dandinong, Australia), H^+^, pH, and Cl^−^ (Ion Selective Meter: “Orion 720“; Orion Research Inc, Boston, USA) and HCO_3_^−^ (titrametrically), and osmolarity (Vapor Pressure Osmometer: “Wescor 5500,” Wescor Inc., Logan, USA) of these urine samples were determined. Other urine parameters, such as glucose, specific gravity, protein, and urine blood were determined using urine reagent test strips (“Uritest 10C,” Uritest Medical Electronic Instrument Co., Ltd., Guilin, China). The color of urine was also noted.

### Assessment of diuretic indices

The percentage of saline load excreted (=volume of urine/volume of saline load × 100), percentage of urinary excretion (=total urinary output/total liquid administered × 100), diuretic action (=urinary output of treated group/urinary output of control group), and diuretic activity (=diuretic action of treated test extract/diuretic action of standard drug) were estimated as described by Nedi *et al*.[11]

Using the data obtained for electrolytes, the saliuretic indices for Na^+^, K^+^, and Cl^−^;[12] aldosterone secretion index (Na^+^/K^+^);[13] thiozide diuretic index (Na^+^ /Cl^−^);[14] carbonic anhydrase inhibition index [Cl^−^ /(Na^+^ + K^+^)];[15] intra- and extra cellular pH regulatory index (HCO_3_^−^/H^+^),[15] and urine alkali index (Na^+^ /H^+^)[14] were computed.

### Assessment of the glomerular filtration rate of experimental rats

Fifty-four rats were randomly divided into 6 equal groups (n = 9/group), fasted (18 h), then hydrated and orally administered with either water or different doses of BTI as treated above: Group (1)–(6). Then, their cumulative urine output was measured after 2 and 24 h post treatment. Blood was also collected at 2 h post-treatment from their tails using aseptic precautions and serum was separated. Creatinine levels in both blood serum and urine were determined colorimetrically (UV–Vis Spectrophotometer: “Varian Carry 50 Scan”, Varian BV, Middelburg, Netherlands) using Quick test kit (Randox Laboratories Ltd., Antrim, UK) and the results of the glomerular filtration rate (GFR) was expressed in terms of clearance of creatinine.[16]

### Evaluation of sub chronic diuretic activity

Fifty-four rats were randomly divided into 6 equal groups (n = 9/group) and orally administered daily for 28 consecutive days, with either water or different doses of BTI as described above. During the treatment, cumulative urine output was measured at day 0, 7, 14, 21, and 28 of fasted (18 h) rats for 6 h of post-treatment.

### Assessment of toxicity effects on experimental rats

Fifty-four rats used for the above chronic treatment were assessed for the development of toxicity signs.[4] During the treatment, each animal was observed daily for overt signs of toxicity (salivation, lacrimation, squinted eyes, writhing, convulsions, tremors, yellowing of fur, and loss of hair), stress (erection of fur and exophthalmia), behavioral abnormalities (impairment of spontaneous movement, climbing, cleaning of face, and ataxia and other postural changes) and aversive behavior (biting and scratching behavior, licking of tail, paw and penis, intense grooming behavior, and vocalization) and diarrhea. On the 29^th^ day, blood was drawn from the tails of these rats under mild ether anesthesia with aseptic precautions and blood serum was used for the assessment of renal toxicity (in terms of urea and creatinine) and liver toxicity [in terms of aspartate transaminase (AST) and alanine transaminase (ALT)].

### Assessment of phytochemicals in black tea

The content of total polyphenols,[17] theaflavins,[18] thearubigins[18] (using UV–Vis Spectrophotometer: “Varian Carry 50 Scan”, Varian BV, Middelburg, Netherlands), catechins[19] and caffeine,[20] (using HPLC: “Perkin Elmer LC 250”, Perkin Elmer Pty Ltd., Glen Waverly, Australia) of black tea were estimated in triplicate samples using standard test methods. Sodium and potassium contents in BTI were determined flame photometrically (“Jencon PFP 7”; Jencons Scientific Limited, Bedfordshire, UK).

### Statistical analysis of the results

Results were expressed as mean ± SEM. Statistical comparisons were made using Minitab 13.3 release: one-way analysis of variance followed by either Tukey’s multiple comparison test, Mann–Whitney *U*- test, or Student’s *t*- test as appropriately. *P* < 0.05 were considered as significant. Linear regression analysis was performed to assess dose-dependencies of the animal experiments.

## RESULTS

Sieve analysis of the samples revealed that, BOPF tea selected was true to the size of the grade (more than 80% of tea particles are in the size range 500 < 800 μm). Tea tasters’ sensory analysis confirmed that the samples used correctly represent the 3 major agroclimatic elevations.

Sri Lankan BOPF grade black tea from three elevations significantly increased cumulative urine output, compared with the control [Table 1]. Furthermore, low (26%), mid (35%), and high (49%) doses of high-grown BTI increased their cumulative urine output dose-dependently (*r*^2^ = 0.9427). A similar increasing pattern was evident when urinary output was evaluated in terms of diuretic action [Table 1]. However, BTI was less potent than furosemide: high-, mid-, and low-grown by 70%, 75%, and 50%, respectively. A similar pattern was evident when the potency was evaluated in terms of diuretic activity [Table 1]. All treatments of BTI of high-grown tea significantly and dose-dependently increased the percentage saline excreted (*r*^2^ = 0.8163) and percentage urinary excretion (*r*^2^ = 0.8285).

**Table 1:** The acute effect of oral administration of different doses of BTI of Sri Lankan BOPF grade black tea from different agroclimatic elevations on cumulative urine output of rats for 6 h

Group No.	Treatment	Dose (mg/kg BW)	Urine volume (mL/100 g/6 h)	% Saline excreted	% Urinary excretion	Diuretic action	Diuretic activity
(1)	Water (control)		2.28 ± 0.203	15.20 ± 1.35	14.25 ± 1.27	-	-
(2)	High-grown	300	2.88 ± 0.236*	19.20 ± 1.57*	18.00 ± 1.47*	1.26	0.50
(3)	High-grown	600	3.08 ± 0.130*	20.54 ± 0.86*	19.25 ± 0.81*	1.35	0.54
(4)	High-grown	2400	3.39 ± 0.376*	22.63 ± 2.50*	21.21 ± 2.35*	1.49	0.59
(5)	Mid-grown	2400	3.30 ± 0.174*	22.03 ± 1.16**	20.65 ± 1.09*	1.45	0.59
(6)	Low-grown	2400	3.84 ± 0.383**	25.62 ± 2.55**	24.02 ± 2.39**	1.68	0.70
(7)	Furosemide	13	5.76 ± 0.264**	38.42 ± 1.76**	36.01 ± 1.65**	2.53	1

BTI: Black tea infusion, BOPF: Broken orange pekoe fannings, BW: Body weight, Values are given as mean ± SEM, n = 9/group

Values are significant at *P* < 0.05* and

*P* < 0.01** compared with control

**Figure 1:**
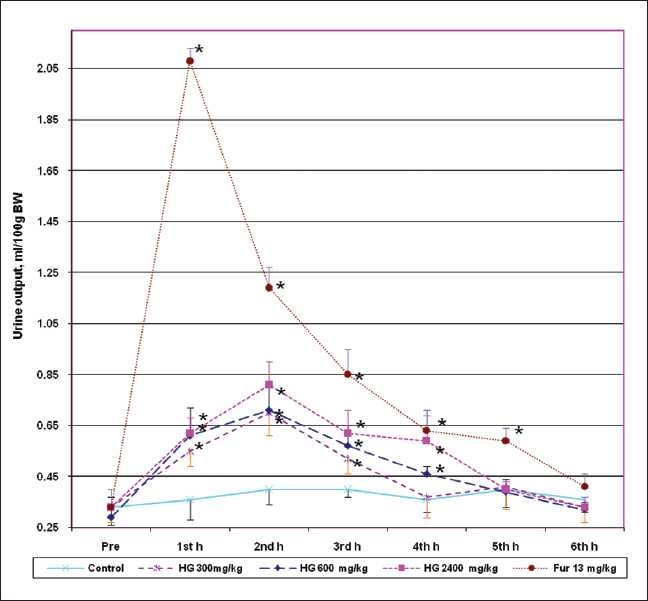
Effect of oral administration of high-grown black tea infusion of Sri Lankan BOPF grade black tea (**Camellia sinensis** L.) with different doses compared with control (water) and positive control (furosemide), on acute diuretic activity in rats. Values are given as Mean ± SEM, n = 9 / group. *values are significant at *P* < 0.05 compared to control

As shown in the Figure 1, all doses except the low dose of high-grown BTI significantly and dose-dependently increased their urinary output from 1^st^ h (*r*^2^ = 0.8073) up to 4^th^ h, peaked at 2^nd^ h (*r*^2^ = 0.8223), whereas the low dose had this effect only up to the 3^rd^ h. In contrast, furosemide had significant diuresis up to 5^th^ h and peaked at 1^st^ h.

The high dose of high-grown BTI significantly increased the frequency of micturition at the 1^st^ h (control vs treatment: 2.11 ± 0.21 vs 3.67 ± 0.31, per hour) and at 2^nd^ h (control vs treatment: 1.33 ± 0.11 vs 6.22 ± 0.71, per hour) of post treatment. Micturition frequency of BTI at 2^nd^ h was more profound (11%) than furosemide (control vs treatment: 1.33 ± 0.11 vs 4.22 ± 0.46, per hour). The total number of urinations was increased significantly and dose-dependently (*r*^2^ = 0.8683) during 6 h post-treatment [control vs treatment: 9.31 ± 0.78 vs 10.44 ± 0.23 (low dose); 11.33 ± 0.76 (mid dose); 15.44 ± 1.11(high dose), per 6 h].

High-grown BTI dose-dependently and significantly increased urinary Na^+^ level (*r*^2^ = 0.9157), which varied with the agroclimatic elevation [control vs treatment: 3728 ± 98 vs 6363 ± 128 (low grown); 6403 ± 72 (mid grown); 6345 ± 153 (high grown) mg/L]. In contrast, high-grown BTI significantly and dose-dependently decreased H^+^ level (*r*^2^ = 0.8991), which varied with the agroclimatic elevation [control vs treatment: 57.28 ± 3.99 vs 1.29 ± 0.19 (low grown); 1.90 ± 0.22 (mid grown); 2.23 ± 0.48 (high grown) × 10^−5^ mmol/L]. On the other hand, other electrolytes measured are not changed, whereas furosemide had significantly altered urinary Na^+^ (by 60%) and K^+^ (by 39%) levels.

As shown, in Table 2, BTI contributed to the increase in sodium saliuretic index in urine dose-dependently (*r*^2^ = 0.9021), and the index was altered with agroclimatic elevation. None of the potassium or chloride saliuretic indices were altered by BTI. As shown, aldosterone secretion index was increased significantly and dose-dependently (*r*^2^ = 0.8972) in high-grown BTI, and this activity varied with agroclimatic elevation of production. The highest aldosterone secretion index was found with mid-grown BTI. An index assessed for thiazide diuretic action, significantly and dose-dependently (*r*^2^ = 0.8705) increased by high-grown BTI. This was altered with agroclimatic elevation, but 46% less potent (high dose) than furosemide. In contrast, carbonic anhydrase inhibition index was significantly impaired with BTI dose-dependently (*r*^2^ = 0.8865) and also changed with agroclimatic elevation, but was 31% less potent (high dose) than furosemide. BTI significantly increased HCO_3_^−^ /H^+^ ratio dose-dependently (*r*^2^ = 0.8925), and the value changed with elevation, but 15% less potent (high dose) than furosemide. BTI caused significantly increased urine alkali index dose-dependently (*r*^2^ = 0.9071). The highest alkali index was evident with low-grown BTI, followed by high-grown and mid-grown but the overall activity was low compared with furosemide [Table 2]. Simultaneously, the urine alkalinity (in terms of pH) also increased dose-dependently (*r*^2^ = 0.8491) but was less alkaline than that of furosemide (data not shown).

**Table 2:** Assessment of diuretic indexes of orally treated rats with different doses of BTI of Sri Lankan BOPF grade black tea from different agroclimatic elevations

Group No.	Treatment	Dose (mg/kg BW)	Saliuretic indices			Aldosterone secretion index (Na^+^/ K^+^)	Thiazide diuretic index (Na^+^/ Cl^−^) × 10^3^	Carbonic anhydrase inhibition index (Cl^-^/Na^+^+K^+^) × 10^−5^	pH regulatory index (HCO_3_^-^/H^+^) × 10^6^	Urine alkali index (Na^+^/ H+) ×l0^8^
			Na^+^	K^+^	Cl^−^					
(1)	Water (control)		—	—	—	1.76 ± 0.059	18.31 ± 0.509	3.49 ± 0.081	0.24 ± 0.02	0.07 ± 0.005
(2)	High-grown	300	1.19	0.95	1.06	2.19 ± 0.059*	20.79 ± 1.040	3.36 ± 0.157	0.85 ± 0.12	0.27 ± 0.037*
(3)	High-grown	600	1.38	1.04	1.08	2.34 ± 0.072*	23.59 ± 0.680*	2.98 ± 0.092*	4.18 ± 0.11*	1.67 ± 0.436*
(4)	High-grown	2400	1.70	1.13	1.07	2.68 ± 0.136*	29.43 ± 1.020**	2.49 ± 0.090*	8.74 ± 1.48**	4.17 ± 0.831**
(5)	Mid-grown	2400	1.72	1.10	1.08	2.78 ± 0.102**	29.30 ± 0.859*	2.53 ± 0.093*	7.98 ± 1.04**	3.81 ± 0.488**
(6)	Low-grown	2400	1.71	1.12	1.08	2.71 ± 0.124*	29.23 ± 1.120*	2.52 ± 0.106*	11.57 ± 1.41**	5.73 ± 0.740**
(7)	Furosemide	13	2.51	1.64	1.04	2.72 ± 0.099*	42.67 ± 1.570**	1.73 ± 0.075*	13.27 ± 1.81**	14.3 ± 13.70**

BTI: Black tea infusion, BOPF: Broken orange pekoe fannings, BW: Body weight, Values are given as mean ± SEM, n = 9/group

Values are significant at *P* < 0.05* and

*P* < 0.01** compared with control

BTI caused increase of GFR significantly and dose-dependently (*r*^2^ = 0.8975), which changed with agroclimatic elevation. Compared with the control, the highest effect was found with low-grown BTI (control vs treated: 0.053 ± 0.008 vs 0.359 ± 0.046 mL/min) followed by mid-grown (control vs 0.242 ± 0.048 mL/min) and high-grown (control vs 0.219 ± 0.024 mL/min). Urinary osmolarity was not altered by any of the BTIs (range: 543.7–547.1 mmol/kg). On the other hand, none of the parameters determined by using urine test strips were altered by BTIs. Besides, BTI did not affect the urine color.

As shown in Table 3, chronic oral administration of BTIs increased the diuretic activity significantly in a dose-dependent manner: day 7 (*r*^2^ = 0.9314), 14 (*r*^2^ = 0.9076), 21 (*r*^2^ = 0.8705) and 28 (*r*^2^ = 0.8825), and they changed with agroclimatic elevation too. On the other hand, compared to pre treatment, significant, time-dependant increased diuresis by BTIs in all elevations was evident when evaluated on weekly basis for 28 days.

**Table 3:** The chronic effect of oral administration (28 days) of different doses of BTI of Sri Lankan BOPF grade black tea from different agroclimatic elevations on diuretic activity of rats

Group No.	Treatment	Dose (mg/kg BW)	Cumulative urine output, mL/100g BW/ 6 h				
			Pre treatment	Chronic treatment			
			3^rd^ day	7^th^ day	14^th^ day	21^st^ day	28^th^ day
(1)	Water (control)		2.15 ± 0.08	2.18 ± 0.10	2.23 ± 0.12	2.26 ± 0.10	2.27 ± 0.10
(2)	High-grown	300	2.12 ± 0.12	2.42 ± 0.21*	2.54 ± 0.12*	2.75 ± 0.14*	2.79 ± 0.13*
(3)	High-grown	600	2.13 ± 0.15	2.83 ± 0.15*	2.95 ± 0.17*	3.07 ± 0.15*	3.09 ± 0.12*
(4)	High-grown	2400	2.15 ± 0.14	2.90 ± 0.14**	3.05 ± 0.15**	3.27 ± 0.16**	3.32 ± 0.11**
(5)	Mid-grown	2400	2.16 ± 0.07	2.91 ± 0.17**	3.16 ± 0.21**	3.32 ± 0.14**	3.36 ± 0.18**
(6)	Low-grown	2400	2.19 ± 0.22	3.06 ± 0.18**	3.21 ± 0.18**	3.37 ± 0.02**	3.39 ± 0.20**

BTI: Black tea infusion, BOPF: Broken orange pekoe fannings, BW: Body weight, Values are given as mean ± SEM, n = 9/group

Values are significant at *P* < 0.05* and

*P* < 0.01** compared with control

The phytochemical analysis of BTIs from low-, mid-, and high-grown, respectively, showed the presence of total polyphenols (195.3 ± 6.40, 220.5 ± 6.67, and 225.8 ± 2.97 g/kg), theaflavins (7.9 ± 0.48, 9.5 ± 0.73, and 10.6 ± 0.45 g/kg), thearubigins (148.6 ± 6.64, 136.6 ± 4.15, and 132.6 ± 2.97 g/kg), total catechins (22.7 ± 0.07, 37.2 ± 3.78, and 51.2 ± 1.73 g/kg), and caffeine (52.6 ± 0.10, 27.5 ± 0.14, and 33.8 ± 0.14 g/kg), The content of sodium (range: 1.2–1.9 g/L) and potassium (range: 17.6–21.0 g/L) in BTIs are found to be in the normal range.

In the toxicity study, any of BTIs did not provoke any overt signs of toxicity, stress, behavioral abnormalities, aversive behavior, or diarrhea. In addition, none of the treated rats died, no renal or liver toxicity was observed.

## DISCUSSION

The results showed that BTI of Sri Lankan BOPF black tea has significant dose-dependent diuretic activity (in terms of cumulative urine output, percentage saline excretion, percentage urinary excretion, and diuretic action index) and natriuretic activity (in terms of increased urine Na^+^ level and sodium saliuretic activity) when tested for 6 h. Diuretic potency of BTI was about 50% less than that of furosemide (in terms of cumulative urine volume and diuretic activity index). Furthermore, diuretic activity of BOPF grade was less potent than that of Dust grade, which has tea particles of smaller size.[4] Thus, the diuretic activity of black tea appears to depend on the size of the tea particles.

The diuretic activity of BTI was dose-dependent, suggesting that the effect was genuine, intrinsic, and casual, and may not have been the result from nonspecific actions. Furthermore, diuretic activity varied with agroclimatic elevations: highest effect was evident with tea obtained from low-grown elevation. This is the first study to show that diuretic activity of black tea is agroclimatic elevation dependant. Also, long-term BTI treatment did not suppress the diuretic activity, an important feature expected of any herbal diuretic agent.

Some herbal diuretics induce diuresis by stimulating the thirst center in the hypothalamus and thereby enhancing the fluid intake.[21] Such a mode of action is unlikely to be operative with BTI since the rats had no access to fluid intake during the 6 h experimental period. Some plant diuretics exert diuresis by having high salt content.[15] Such a nonspecific mechanism is unlikely to be operative here since BTI had extremely low content of Na^+^ and K^+^ ions; in spite of high Na^+^ level in urine, BTI did not alter the osmolarity and specific gravity of urine of treated rats. Thus, the diuretic effect is not related to an osmotic type of mechanism.[12] Furthermore, osmotic diuretics are orally inactive and usually given intravenously.[22] The diuretic effect of BTI is also unlikely to be due to an impairment of the action of antidiuretic hormone because such an impairment causes polyuria with low osmolarity.[23] Loop diuretics causes increase in urinary Na^+^ and Cl^−^ levels.[14] But BTI increased urinary Na^+^ level alone, suggesting that diuretic activity of BTI is different from furosemide-like loop diuretics. The onset of BTI-induced diuresis was always rapid (within 1 h) and it did not reduce the urinary K^+^ level. Collectively, these observations indicate that BTI is not acting as a potassium sparing diuretic.[24]

BTI made from all agroclimatic elevations significantly increased the urinary Na^+^ /Cl^−^ ratio (thiazide diuretic index).[25] Thiazide diuretics inhibit the Na^+^ /Cl^−^ symporter (co-transporter system) in the distal convoluted tubule, by competing for the Cl^−^ binding sites, and increasing the excretion of Na^+^ by inhibiting Na^+^ re-absorption.[25] Furthermore, like other thiazide diuretics,[26] BTI did not alter urinary Ca^2+^ level. Therefore, this study show, for the first time that, BTI has thiazide type of diuretic action. BTI of all agroclimatic elevations significantly decreased urinary Cl^−^ /(Na^+^ + K^+^) ratio (index for increased carbonic anhydrase inhibitory activity).[15] On the other hand, BTI of all elevations significantly increased urinary HCO_3_^−^ / H^+^ ratio (cellular pH regulatory index).[15] Collectively this indicates an inhibition of carbonic anhydrase activity. Thus, one of the mechanisms of diuretic activity of BTI may be mediated via inhibition of carbonic anhydrase activity. Interestingly, this mechanism of diuretic activity has not been previously reported for any kind of tea. BTI caused to increase urinary Na^+^ level without changing urinary K^+^ level and BTI significantly increased urinary Na^+^ /K^+^ ratio (aldosterone secretion index).[13] This indicates a blocking of aldosterone action leading to suppressing the release of K^+^ ions from plasma,[27,28] resulting in the slight alkalization of urine[22] and unchanged osmolarity of excreted urine.[29] This could be one of the mechanisms via which BTI induces diuresis.

In this study, marked natriuresis (in terms of increased urinary Na^+^ level and sodium saliuretic index) was evident possibly because of the inhibition of Na^+^ resorption in the nephron,[4] thereby increasing the urinary output. This could be attributed to the caffeine (a methylxanthene) present in black tea, which is known to inhibit Na^+^ resorption in nephrons.[30] Among 3 major agroclimatic elevations, the highest diuresis was evident with low-grown BTI where the caffeine content was found to be significantly high. Furthermore, decaffeination of black tea is shown to almost completely abolish its diuretic activity.[4] In this study, an increase in GFR was evident (in terms of creatinine clearance)[16] with BTI treatment. This mechanism too can contribute to BTI-induced diuresis because caffeine in black tea has been shown to increase renal blood flow and cardiac output.[30] Some plant extracts contain flavonoids which, due to their anti-inflammatory properties, promote diuresis.[31] BTI also possesses anti-inflammatory activity,[32] and therefore, a possibility exists that, one of the mechanisms of diuretic activity of BTI may result from this action.

The present study showed that the content of some phytochemicals in black tea: polyphenols, majority of catechins, and caffeine vary with the agroclimatic elevation. Catechins and caffeine found in plant extracts have been reported to promote diuretic activity.[33] Furthermore, the diuretic activity of BTI can vary with the agroclimatic elevation as shown in this study. The rapid onset of diuretic activity of BTI (at 1^st^ h) indicates that the diuresis is mediated via quick absorption of phytochemicals from the gastrointestinal tract[34] and is not due to secondary metabolites of the phytochemicals.[31] Unlike some catechins, caffeine is rapidly absorbed from the stomach and intestine and known to alter intestinal absorptive and secretary functions[35] and may be one of the reasons for rapid onset (within 1 h) of the diuretic activity. Catechins have a much shorter half-life in plasma (only 2–3 h) than the other flavonoids and most metabolites are excreted during the first 4 h.[31] This may be one of the reasons for the short duration of diuretic action of BTI. The highest frequency of micturition was observed (at 2^nd^ h) during the 6 h period, and this may at least or partly account for the peak urinary output at 2 h, observed in this study.

Toxicity studies showed that BTI of all elevations was well tolerated [in terms of overt signs of toxicity, renotoxicity (in terms of serum urea and creatinine levels), hepatotoxicity (in terms of serum ALT and AST). Furthermore, no mortality was evident during the toxicity studies. These observations suggest that BTI is safe for regular consumption.

In conclusion, this study shows for the first time, that BTI of Sri Lankan BOPF grade black tea manufactured from **Camellia sinensis** L. plant possesses pharmacologically safe, mild oral diuretic activity, which is mediated via multiple mechanisms: increased urinary Na^+^ output, inhibition of aldosterone secretion, inhibition of carbonic anhydrase activity, and through thiazide-like diuretic action. The diuretic potential varied with the agroclimatic elevation, the highest being recorded with the low-grown tea. This study also indicates that Sri Lankan BOPF grade teas of all agroclimatic elevations could be used by traditional practitioners to promote urinary flushing.
